# *Sensors* Best Paper Award 2011

**DOI:** 10.3390/s110101243

**Published:** 2011-01-25

**Authors:** Ophelia Han

**Affiliations:** MDPI AG, Postfach, CH-4005 Basel, Switzerland and MDPI Branch Office, Beijing, China; Tel.: +86-10-590-110-09; Fax: +86-10-590-110-89; E-Mail: ophelia.han@mdpi.com

With the start of 2011, *Sensors* is instituting an annual award to recognize outstanding papers related to sensing technologies and applications that meet the aims, scope and high standards of this journal. We are pleased to announce the first “*Sensors* Best Paper Award” for 2011. Nominations were solicited from the Section Editor-in-Chiefs of *Sensors*, with all papers published in 2005 eligible for consideration. The following three papers were awarded:

**1^st^ Prize**
Yajing Yin, Yafen Lü, Ping Wu and Chenxin Cai *
  Direct Electrochemistry of Redox Proteins and Enzymes Promoted by Carbon Nanotubes.
  *Sensors* **2005**, *5*(4), 220–234; doi:10.3390/s5040220
  Available online: http://www.mdpi.com/1424-8220/5/4/220/

**2^nd^ Prize**
Vojtech Adam Adam, Josef Zehnalek, Jitka Petrlova, David Potesil, Bernd Sures, Libuse Trnkova, Frantisek Jelen, Jan Vitecek and Rene Kizek *
  Phytochelatin Modified Electrode Surface as a Sensitive Heavy-Metal Ion Biosensor.
  *Sensors* **2005**, *5*(1), 70–84; doi:10.3390/s5010070
  Available online: http://www.mdpi.com/1424-8220/5/1/70/

**3^rd^ Prize**
**Kevin A. Delin, Shannon P. Jackson, David W. Johnson, Scott C. Burleigh, Richard R. Woodrow, J. M. McAuley, James M. Dohm, Felipe Ip, Ty P. Ferré, Dale F. Rucker and Victor R. Baker**
  Environmental Studies with the Sensor Web: Principles and Practice.
  *Sensors* **2005**, *5*(1), 103–117; doi:10.3390/s5010103
  Available online: http://www.mdpi.com/1424-8220/5/1/103/

The prize awarding committee merits the article “Direct Electrochemistry of Redox Proteins and Enzymes Promoted by Carbon Nanotubes” for reporting “…excellent science on a timely topic…” and acknowledges the article “Phytochelatin Modified Electrode Surface as a Sensitive Heavy-Metal Ion Biosensor” as a “…nicely done paper with a new approach…”.

These three exceptional papers are valuable contributions to *Sensors* and the sensing field. On behalf of the Prize Awarding Committee and the Editorial Board of *Sensors*, we would like to congratulate these three teams for their excellent work. In recognition for their accomplishment, Drs. Chenxin Cai, Rene Kizek and Kevin A. Delin will receive a prize of 1000 CHF, 500 CHF, and 300 CHF, respectively, and the privilege to publish an additional paper free of charge in open access format in *Sensors,* after the usual peer-review procedure.

**Prize Awarding Committee**
*Editor-in-Chief*, Section ‘Physical Sensors’
  **Prof. Dr. Craig A. Grimes**
  Department of Electrical Engineering & Materials Research Institute, 217 Materials Research Laboratory, the Pennsylvania State University, University Park, PA 16802, USA
  E-Mail: cgrimes@engr.psu.edu

*Editor-in-Chief*, Section ‘Remote Sensors’
  **Dr. Assefa M. Melesse**
  Department of Environmental Studies, ECS 339, Florida International University, 11200 SW 8th Street, Miami, FL 33199, USA
  E-Mail: assefa.melesse@fiu.edu

*Editor-in-Chief*, Section ‘Chemical Sensors’
  **Prof. Dr. W. Rudolf Seitz**
  Analytical Chemistry, Department of Chemistry, University of New Hampshire, Durham, NH 03824-3598, USA
  E-Mail: wrs@cisunix.unh.edu

*Editor-in-Chief*, Section ‘Biosensors’
  **Dr. Alexander Star**
  Department of Chemistry, University of Pittsburgh, 219 Parkman Avenue, Pittsburgh, PA 15260, USA
  E-Mail: astar@pitt.edu

Managing Editor
  **Dr. Ophelia Han**
  MDPI Beijing Office, Liyuanbeijie Road 186, Suite 307, Liyuan Town, Tongzhou District, 101101 Beijing, China
  E-Mail: ophelia.han@mdpi.com

